# Insulin-Like Growth Factor (IGF) Binding Protein-2, Independently of IGF-1, Induces GLUT-4 Translocation and Glucose Uptake in 3T3-L1 Adipocytes

**DOI:** 10.1155/2017/3035184

**Published:** 2017-12-20

**Authors:** Biruhalem Assefa, Ayman M. Mahmoud, Andreas F. H. Pfeiffer, Andreas L. Birkenfeld, Joachim Spranger, Ayman M. Arafat

**Affiliations:** ^1^Department of Endocrinology, Diabetes and Nutrition, Charité-University Medicine Berlin, Berlin, Germany; ^2^Department of Endocrinology, Diabetes and Nutrition at the Center for Cardiovascular Research (CCR), Charité-University Medicine Berlin, Berlin, Germany; ^3^Division of Physiology, Department of Zoology, Faculty of Science, Beni-Suef University, Beni-Suef, Egypt; ^4^Department of Clinical Nutrition, German Institute of Human Nutrition Potsdam-Rehbrücke, Nuthetal, Germany; ^5^Section of Metabolic Vascular Medicine, Medical Clinic III, and Paul Langerhans Institute Dresden (PLID), Dresden University of Technology, Dresden, Germany; ^6^Division of Diabetes & Nutritional Sciences, Faculty of Life Sciences & Medicine, King's College London, London, UK; ^7^Department of Endocrinology, Diabetes and Nutrition at the Experimental and Clinical Research Centre (ECRC), Charité-University Medicine Berlin and Max-Delbrück Center Berlin-Buch, Berlin, Germany

## Abstract

Insulin-like growth factor binding protein-2 (IGFBP-2) is the predominant IGF binding protein produced during adipogenesis and is known to increase the insulin-stimulated glucose uptake (GU) in myotubes. We investigated the IGFBP-2-induced changes in basal and insulin-stimulated GU in adipocytes and the underlying mechanisms. We further determined the role of insulin and IGF-1 receptors in mediating the IGFBP-2 and the impact of IGFBP-2 on the IGF-1-induced GU. Fully differentiated 3T3-L1 adipocytes were treated with IGFBP-2 in the presence and absence of insulin and IGF-1. Insulin, IGF-1, and IGFBP-2 induced a dose-dependent increase in GU. IGFBP-2 increased the insulin-induced GU after long-term incubation. The IGFBP-2-induced impact on GU was neither affected by insulin or IGF-1 receptor blockage nor by insulin receptor knockdown. IGFBP-2 significantly increased the phosphorylation of PI3K, Akt, AMPK, TBC1D1, and PKC*ζ*/*λ* and induced GLUT-4 translocation. Moreover, inhibition of PI3K and AMPK significantly reduced IGFBP-2-stimulated GU. In conclusion, IGFBP-2 stimulates GU in 3T3-L1 adipocytes through activation of PI3K/Akt, AMPK/TBC1D1, and PI3K/PKC*ζ*/*λ*/GLUT-4 signaling. The stimulatory effect of IGFBP-2 on GU is independent of its binding to IGF-1 and is possibly not mediated through the insulin or IGF-1 receptor. This study highlights the potential role of IGFBP-2 in glucose metabolism.

## 1. Introduction

Insulin-like growth factor-1 (IGF-1) bears structural homology with proinsulin [1] and plays a key role in the proliferation and differentiation of adipocytes [2]. *In vitro,* it is known to exert mitogenic effects at nanomolar concentrations [3] and to induce insulin-like metabolic effects in both muscle and adipose tissues [4]. The production and secretion of IGF-1 is affected by age, nutritional status, and other hormones [5]. Because of the ability of insulin to induce hepatic growth hormone (GH) receptor gene expression [6] and protein abundance [7], the GH-induced synthesis and release of IGF-1 is highly dependent on the hepatic insulin sensitivity. This interplay among GH, insulin, and IGF-1 is of key importance for metabolic and growth regulation [8].

The bioavailability of IGFs is regulated by a family of seven structurally conserved binding proteins (IGFBPs) [9–11]. These IGFBPs bind IGF-1 and IGF-2 but not insulin [12]. The IGF-1 independent role of IGFBPs in growth and metabolism has also been reported at least *in vitro* [13, 14]. IGFBP-2 is the predominant binding protein produced during adipogenesis of white preadipocytes [15]. Both inhibitory and stimulatory effects of IGFBP-2 on the cellular actions of IGF-1 and IGF-2 have been reported [16, 17]. IGFBP-2 is reported to be a key regulator of metabolic diseases, such as diabetes and obesity. Low IGFBP-2 has been shown to be associated with higher fasting glucose levels and reduced insulin sensitivity suggesting it as a biomarker for identification of insulin-resistant individuals [18]. Moreover, IGFBP-2 gene expression was downregulated in visceral white adipose tissue of mice and its circulating levels were reduced in obese ob/ob, db/db, and high fat-fed mice [19]. Low levels of circulating IGFBP-2 have also been reported in obese adults [20] and children [21].

Wheatcroft and colleagues demonstrated that IGFBP-2 overexpression conferring protection against age-associated decline in insulin sensitivity in mice [22]. Moreover, the leptin-induced overexpression of IGFBP-2 has been shown to reverse diabetes in insulin-resistant obese mice and hyperinsulinemic clamp studies showed a threefold improvement in hepatic insulin sensitivity following IGFBP-2 treatment of ob/ob mice [23]. However, only few information exists to date regarding the mechanisms underlying the positive IGFBP-2-induced impact on glucose metabolism. Indeed, IGFBP-2 has been shown to increase the insulin-stimulated glucose uptake in myotubes [24] but nothing is known about its impact on glucose uptake in adipocytes with respect to the insulin or IGF-1-induced effects. We, therefore, aimed to investigate the IGFBP-2-induced changes in both basal and insulin-stimulated glucose uptake in 3T3-L1 adipocytes and the underlying mechanisms. We further investigated the role of insulin and IGF-1 receptors in mediating the IGFBP-2 and even the impact of IGFBP-2 on the IGF-1-induced improvement in glucose uptake.

## 2. Materials and Methods

### 2.1. Reagents, Hormones, and Antibodies

IGF-1 and IGF-1 Long R3 (IGF-1 LR3) were purchased from BioVision Inc. (Milpitas, CA, USA). IGFBP-2, Dulbecco's Modified Eagle Medium (DMEM), penicillin/streptomycin, and fetal bovine serum (FBS) were purchased from Biochrom AG (Berlin, Germany). Insulin, dexamethasone, LY294002, and picropodophyllin (PPP) were supplied by Sigma-Aldrich (Darmstadt, Germany). 3-Isobutyl-1-methylxanthine (IBMX), S961, wortmannin, and Compound C were purchased from Biomol GmbH (Hamburg, Germany), Phoenix Biotech (Beijing, China), Merck Chemicals (Darmstadt, Germany), and BIOZOL Diagnostica Vertrieb (Eching, Germany), respectively. RevertAid First Strand cDNA Synthesis Kit, SYBR Green master mix, Bicinchoninic Acid (BCA) protein assay kit, and ECL reagent were supplied by Thermo Fisher Scientific (Dreieich, Germany). DNA primers were purchased from Eurogentec Deutschland GmbH (Köln, Germany). All other chemicals were supplied by Sigma-Aldrich (Darmstadt, Germany).

### 2.2. Cell Culture

The murine fibroblast cell line 3T3-L1 (ATCC, Manassas, VA, USA) was cultured in DMEM supplemented with 4.5 g/L glucose, 10% fetal bovine serum (FBS), 4 mM glutamine, 50 U/ml penicillin, and 50 *μ*g/ml streptomycin until confluence. The cells were incubated to differentiate into adipocytes following the method of Woody et al. [25] with slight modifications. Briefly, 2 days postconfluence, cells were treated with 0.5 mM IBMX, 1 *μ*M dexamethasone, and 1 *μ*M insulin supplemented DMEM for 2 days. The cells were then maintained in 1 *μ*M insulin-supplemented growth medium for 3 days and in growth medium for 4 days prior to experiments.

### 2.3. Transfection of Insulin Receptor (INSR) siRNAs

Differentiated 3T3-L1 adipocytes were transfected with control or INSR specific siRNA (validated siRNA from Dharmacon) using Lipofectamine RNAiMAX (Invitrogen) for 72 h. The efficiency of transfection was assessed by using qPCR and Western blot.

### 2.4. Measurement of Glucose Uptake

Glucose uptake was assayed using the method described by Yamamoto et al. [26]. Briefly, differentiated 3T3-L1 adipocytes were serum starved for 4 h followed by incubation in D-glucose free DMEM for 1 h. The adipocytes were washed with PBS (pH 7.4) and then incubated for 30 min in Krebs-Ringer bicarbonate buffer (KRBP) with different concentrations of insulin, IGF-1, IGF-1 LR3 and/or IGFBP-2. IGF1 LR3 is an analogue of IGF-1 in which the glutamic acid at carbon 3 (Glu3) is replaced by arginine and contains 13 extra amino acids to the N-terminus. It has a very low affinity towards IGFBPs as compared to IGF-1 [27]. The rationale for using IGF1-LR3 was to investigate whether IGFBP-2 is able to impact the IGF-1-induced increase in glucose uptake regardless of its binding to IGF-1 itself. Had IGFBP-2 exerted additive effect on the IGF-1-induced glucose uptake, it would be imperative to scrutinize the observed effect as due to binding or other means. In some experiments, the adipocytes were incubated with 100 nM S961 (INSR blocker) for 2 h, 60 nM PPP (IGF-1 receptor blocker) for 4 h, 100 *μ*M LY294002 (PI3K inhibitor) for 1 h, 200 nM wortmannin (PI3K inhibitor) for 30 min, or 200 *μ*M Compound C (AMPK inhibitor) for 20 min before the treatment. The adipocytes were treated with [^3^H] 2-Deoxy-D-glucose (0.5 *μ*Ci/ml in HEPES) for 10 min at room temperature (RT) and then washed with PBS. Thereafter, the cells were lysed in 50 mM NaOH/1% Triton X-100 for scintillation counting using a liquid scintillation counter (PerkinElmer Wallac GmbH, Freiburg, Germany). Each experiment was performed with three technical replicates and total number of experiments was three.

### 2.5. Quantitative Reverse Transcriptase-Polymerase Chain Reaction (qRT-PCR) Analysis

Total RNA was isolated from 3T3-L1 adipocytes using TRizol reagent and was treated with DNase I. RNA was quantified at 260 nm using a NanoDrop (Peqlab Biotechnologie, Erlangen, Germany) and samples with A260/A280 ratios < 1.8 were discarded. 1 *μ*g RNA was reverse transcribed into cDNA using RevertAid First Strand cDNA Synthesis Kit (Thermo Fisher Scientific, Dreieich, Germany). cDNA was amplified using SYBR Green master mix (Thermo Fisher Scientific, Dreieich, Germany) with the primers set outlined in Table 1 and with the following conditions: initial denaturation step at 95°C for 10 min, followed by 40 cycles of 15 sec at 95°C, 60 sec at annealing temperature of respective primer, and 60 sec at 72°C for extension. Melting curve analysis was used to assess the quality of PCR products and the cycle threshold (CT) values were analyzed using the 2^−ΔΔCt^ method. Data were normalized to 36B4 and presented as % of control.

### 2.6. Preparation of a Plasma Membrane Fraction for Glucose Transporter (GLUT)-4 Translocation Assay

The amount of GLUT-4 in the cell membranes was determined using subcellular fractionation [28] followed by Western blotting analysis. Adipocytes were washed 3 times with ice-cold HEPES-EDTA-sucrose (HES) buffer (pH 7.4) containing proteinase inhibitors. The cell suspension was homogenized by passing through 22-gauge needles 10 times on ice. The homogenate was centrifuged at 16000*g* for 30 min at 4°C, and the pellet was suspended in HES buffer followed by centrifugation at 16000*g* for 30 min at 4°C. The pellet was resuspended in HES buffer, layered on the top of sucrose cushion (38.5% sucrose, 20 mM HEPES and 1 mM EDTA, pH 7) in 1 : 1 volume ratio, and centrifuged at 100000*g* for 1 h at 4°C. The plasma membrane fraction (middle layer) was carefully collected and centrifuged at 40000*g* for 20 min at 4°C. The pellet was used to determine the amount of GLUT-4 using Western blotting.

### 2.7. Western Blot Analysis

Treated 3T3-L1 adipocytes were lysed in RIPA buffer supplemented with inhibitors for proteinases and phosphatases. For the GLUT-4 translocation experiments, the samples were lysed in a specific buffer (10 mM Tris-HCl [pH 7.2], 150 mM NaCl, 5 mM EDTA, 1% Triton X-100, 1% sodium deoxycholate, and 0.1% SDS) supplemented with proteinase and phosphatase inhibitors. The protein content in the samples was measured by BCA assay kit. Proteins (30–50 *μ*g) were denatured and resolved in 10% SDS/PAGE and transferred to nitrocellulose membranes. For GLUT-4, 8% SDS/PAGE was used. Blots were blocked for 1 h and probed with 1 : 1000 diluted primary antibodies for phosphoinositide 3-kinase (PI3K) p85, phospho-(Tyr) PI3K p85, protein kinase B (Akt), phospho-Akt (Ser473), AMP-activated protein kinase alpha (AMPK*α*), phospho-AMPK*α* (Thr172), atypical protein kinase (PKC*ζ*), phospho-PKC*ζ*/*λ* (Thr410/403), TBC1D1 (tre-2/USP6, BUB2, cdc16 domain family member 1), phospho-TBC1D1 (Ser237), and GAPDH overnight at 4°C and with GLUT-4 antibody and Na^+^/K^+^-ATPase for 1 h at RT. The blots were washed and incubated with 1 : 2000 diluted corresponding horseradish peroxidase- (HRP-) labeled secondary antibodies. Details of the used antibodies are listed in Table 2. After washing, the membranes were developed with an ECL reagent and visualized and densitometry analysis using Image Lab™ Software (Bio-Rad Laboratories GmbH, Munich, Germany) was used to quantify protein signal.

### 2.8. Statistical Analysis

Data were analyzed for statistical significance by one-way analysis of variance (ANOVA) with Tukey's post hoc test using GraphPad Prism 5 (La Jolla, CA, USA). The results were presented as means ± standard error of the mean (SEM) with values of *P* < 0.05 were considered significant.

## 3. Results

### 3.1. Effect of IGFBP-2 on Basal as well as Insulin and IGF-1-Induced Increase in Glucose Uptake in 3T3-L1 Adipocytes

**Figure 1:**
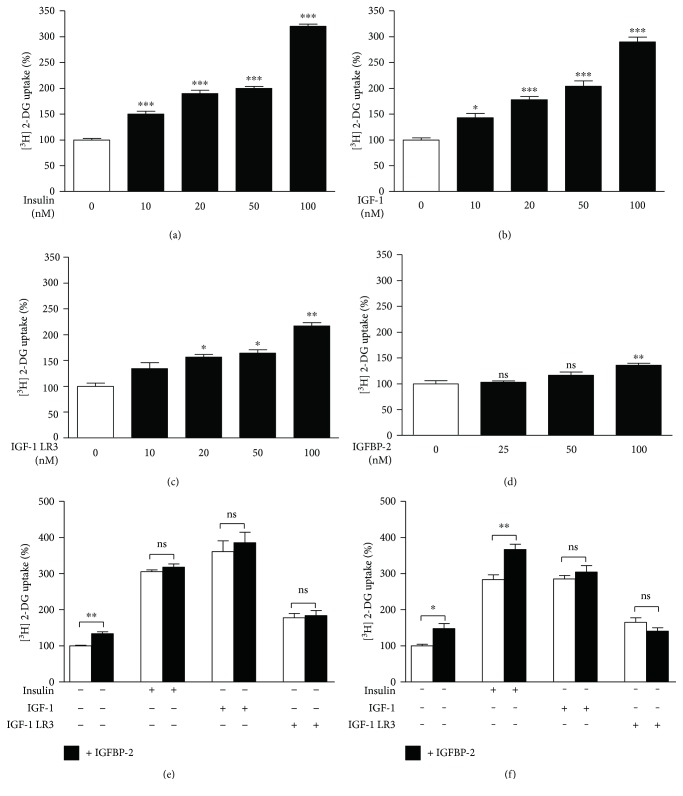
Effects of IGFBP-2 on insulin and IGF-1-stimulated glucose uptake in 3T3-L1 adipocytes. (a–d) Dose-dependent effects of insulin, IGF-1, IGF-1 LR3, and IGFBP-2 on glucose uptake in 3T3-L1 adipocytes. ^∗^*P* < 0.05, ^∗∗^*P* < 0.01, and ^∗∗∗^*P* < 0.001 versus control. (e and f) Effect of IGFBP-2 (100 nM) (black bars) on basal and insulin (20 nM), IGF-1 (20 nM), and IGF-1 LR3 (20 nM)-induced glucose uptake (white bars) after short- and long-term incubations. ^∗^*P* < 0.05 and ^∗∗^*P* < 0.01. Each experiment was performed with three technical replicates and total number of experiments was three. The glucose uptake values are percentage of the controls. The results are presented as mean ± SEM.

To study the effect of insulin, IGF-1, IGF-1 LR3, and IGFBP-2 on glucose uptake in 3T3-L1 adipocytes, the cells were incubated with different concentrations of all tested agents for 30 min and [^3^H] 2-Deoxy-D-glucose uptake was assayed. Insulin and IGF-1 were able to exert statistically significant effects on glucose uptake. As represented in Figure 1(a), different concentrations of insulin (10, 20, 50, and 100 nM) were able to exert a significant (*P* < 0.001) increase in glucose uptake. IGF-1 as well produced a significant increase in glucose uptake at either 10 nM (*P* < 0.05) or higher concentrations (*P* < 0.001) as depicted in Figure 1(b).

Treatment of the cells with the lengthened analogue of IGF-1, IGF-1 LR3, induced significant increase in glucose uptake first at higher concentrations (20, 50, and 100 nM) (Figure 1(c)). Similarly, IGFBP-2 was able to significantly (*P* < 0.01) increase glucose uptake in adipocytes first at a concentration of 100 nM as compared to control cells (Figure 1(d)).

Next, we determined both the short- and long-term impact of IGFBP-2 on insulin, IGF-1, and IGF-1 LR3-induced glucose uptake in adipocytes.

Short-term incubation of the cells with 1 : 1 stoichiometric ratio of IGFBP-2 and either insulin, IGF-1, or IGF-1 LR3 for 30 min resulted in no additive increase in glucose uptake when compared to insulin, IGF-1, or IGF-1 LR3 alone (Figure 1(e)).

Long-term incubation (24 h) of the cells with IGFBP-2 significantly (*P* < 0.05) increased basal glucose uptake and exerted an additive effect (*P* < 0.01) on insulin-stimulated glucose uptake. However, adipocytes treated with IGFBP-2 for 24 h showed nonsignificant changes in either IGF-1 or IGF-1 LR3-induced glucose uptake (Figure 1(f)).

### 3.2. The IGFBP-2-Induced Impact on Glucose Uptake Is Not Mediated through the Activation of Insulin or IGF-1 Receptor

To investigate whether the stimulatory effect of IGFBP-2 on glucose uptake is mediated through its binding to insulin or IGF-1 receptors, we incubated 3T3-L1 adipocytes with either insulin receptor blocker (S961) or IGF-1 receptor blocker (PPP).

**Figure 2:**
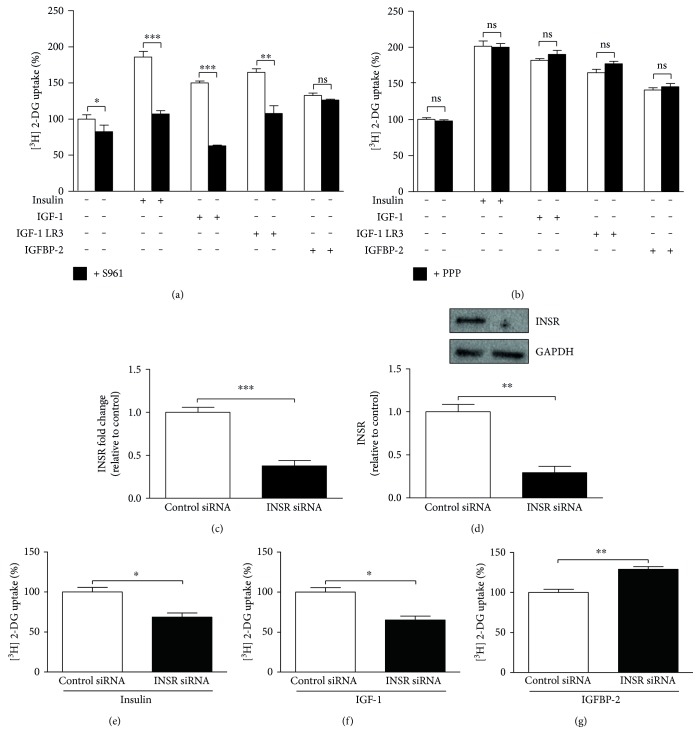
IGFBP-2 stimulates glucose uptake in insulin receptor and IGF-1 receptor-independent mechanism. (a) Effect of the insulin receptor (INSR) blocker S961 (black bars) on basal and insulin, IGF-1, IGF-1 LR3, and IGFBP-2-induced glucose uptake (white bars). (b) Effect of the IGF-1 receptor blocker PPP (black bars) on basal and insulin, IGF-1, IGF-1 LR3, and IGFBP-2 induced glucose uptake (white bars). Differentiated 3T3-L1 adipocytes were incubated with 100 nM S961 for 2 h or 60 nM PPP for 4 h before treatment with insulin, IGF-1, IGF-1 LR3, or IGFBP-2 for 30 min. (c and d) Relative mRNA expression, normalized to 36B4, and Western blot analysis of INSR following siRNA transfection, respectively. (e, f, and g) Effect of 30 min treatment with insulin, IGF-1, and IGFBP-2 on glucose uptake in control siRNA and INSR siRNA transfected 3T3-L1 adipocytes. Each experiment was performed with three technical replicates and total number of experiments was three. The glucose uptake values are percentage of the controls. The results are presented as mean ± SEM. ^∗^*P* < 0.05, ^∗∗^*P* < 0.01, and ^∗∗∗^*P* < 0.001.

3T3-L1 adipocytes incubated for 2 h with S961 showed a significant (*P* < 0.05) decrease in basal glucose uptake when compared with the control cells (Figure 2(a)). The insulin receptor blocker S961 significantly reduced insulin (*P* < 0.001), IGF-1 (*P* < 0.001), and IGF-1 LR3 (*P* < 0.01) and stimulated glucose uptake, whereas no impact (*P* > 0.05) of such treatment on IGFBP-2-stimulated glucose uptake was seen.

When compared with S961, the IGF-1 receptor blocker PPP was not able to induce any significant (*P* > 0.05) effect on glucose uptake in adipocytes neither under basal conditions nor following stimulation with IGF-1, IGF-1 LR3, or IGFBP-2 (Figure 2(b)).

In 3T3-L1 adipocytes transfected with control or INSR-specific siRNA (Figures 2(c) and 2(d)), insulin (Figure 2(e)) and IGF-1-stimulated glucose uptake (Figure 2(f)) were significantly (*P* < 0.05) reduced, whereas INSR knockdown potentiated the effect of IGFBP-2 on glucose uptake when compared with the control cells (*P* < 0.01) (Figure 2(g)).

### 3.3. IGFBP-2 Stimulates Glucose Uptake in a PI3K-Dependent Manner

**Figure 3:**
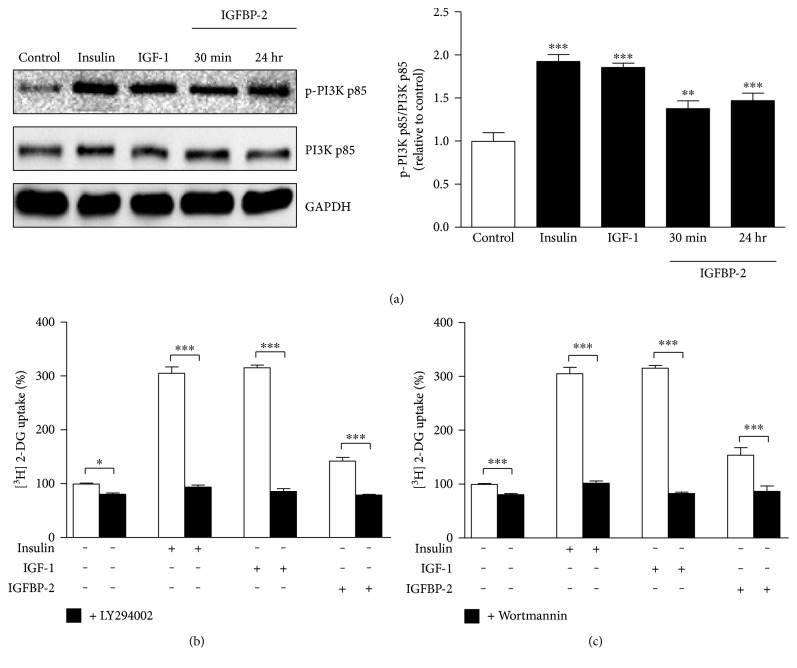
IGFBP-2 stimulates glucose uptake in a PI3K-dependent manner. (a) Insulin, IGF-1, and IGFBP-2 increase the phosphorylation of PI3K. 3T3-L1 cells were cultured and differentiated in 24-well plates for glucose uptake assay and in 6-well plates for Western blotting analysis. The results are presented as mean ± SEM. ^∗^*P* < 0.05, ^∗∗^*P* < 0.01, and ^∗∗∗^*P* < 0.001 versus control. (b and c) The PI3K inhibitors, LY294002 and wortmannin (black bars), significantly reduce basal as well as insulin, IGF-1, and IGFBP-2-induced glucose uptake (white bars). Differentiated 3T3-L1 adipocytes were incubated with 100 *μ*M LY294002 for 1 h or 200 nM wortmannin for 30 min before treatment with insulin, IGF-1, or IGFBP-2 for 30 min. The results are presented as mean ± SEM. ^∗∗∗^*P* < 0.001. Each experiment was performed with three technical replicates and total number of experiments was three.

Adipocytes treated with insulin and IGF-1 for 30 min exhibited significant (*P* < 0.001) increase in PI3K phosphorylation when compared with the control cells. Similarly, IGFBP-2 induced a significant increase in PI3K phosphorylation in 3T3-L1 adipocytes treated for either 30 min (*P* < 0.01) or 24 hr (*P* < 0.001) (Figure 3(a)).

The effect of PI3K inhibitors (LY294002 and wortmannin) on glucose uptake was investigated to further determine the role of PI3K in mediating the IGFBP-2-stimulated glucose uptake in 3T3-L1 adipocytes. Treatment of the adipocytes with either LY294002 (Figure 3(b)) or wortmannin (Figure 3(c)) induced a significant decline in basal as well as insulin-, IGF-1-, and IGFBP-2-stimulated glucose uptake (*P* < 0.001).

### 3.4. IGFBP-2 Induces Akt and AMPK Phosphorylation and the Subsequent Increase in GLUT-4 Translocation in a PI3K-Dependent Manner

**Figure 4:**
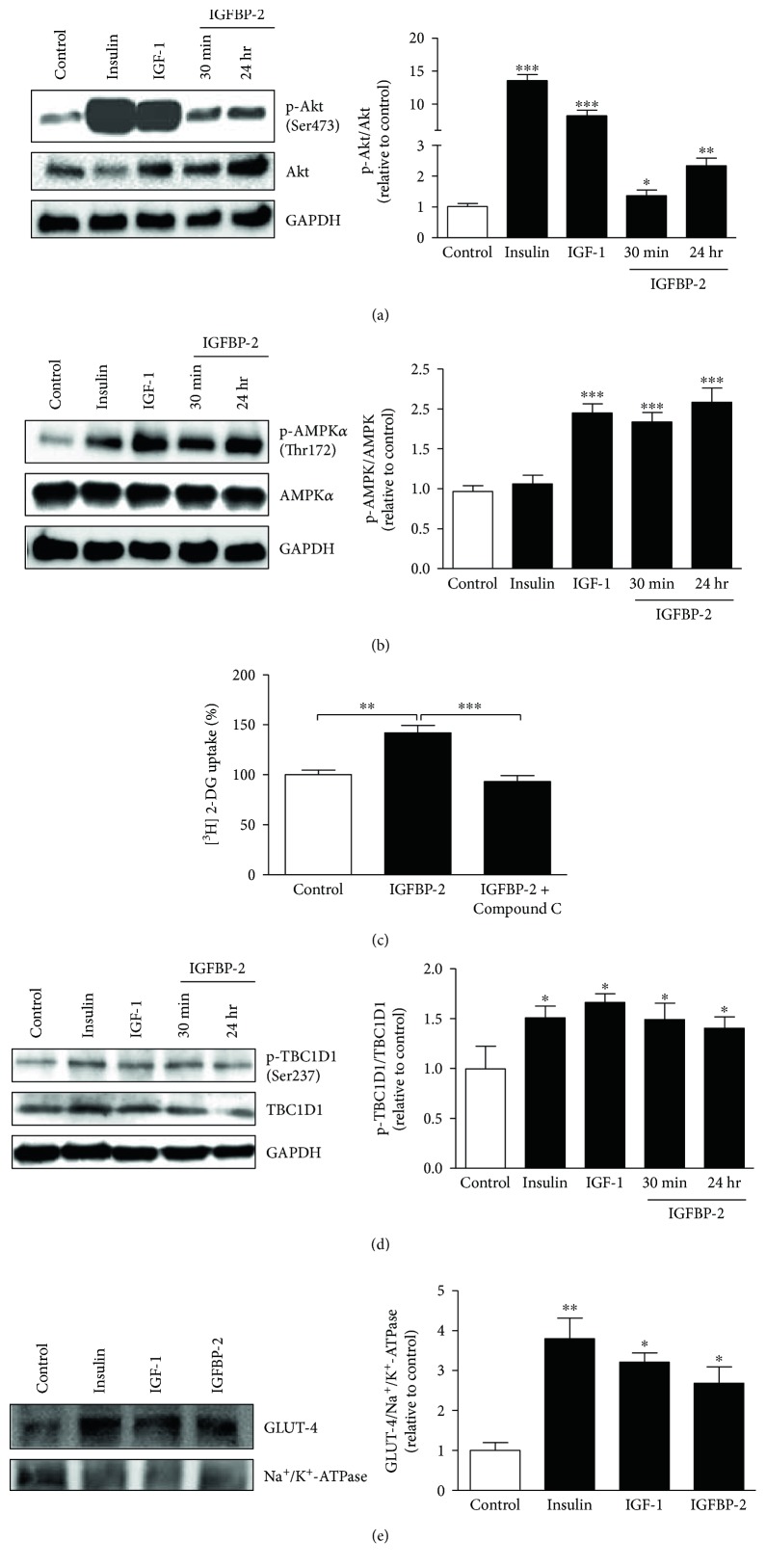
IGFBP-2 stimulates Akt and AMPK activation and thus increased GLUT-4 translocation. (a) Insulin, IGF-1, and IGFBP-2 significantly increase Akt Ser473 phosphorylation. (b) IGF-1 and IGFBP-2 but not insulin increase AMPK Thr172 phosphorylation. ^∗^*P* < 0.05, ^∗∗^*P* < 0.01, and ^∗∗∗^*P* < 0.001 versus control. (c) The AMPK inhibitor, Compound C, abolishes IGFBP-2-induced glucose uptake in 3T3-L1 adipocytes. Differentiated 3T3-L1 adipocytes were incubated with 200 *μ*M Compound C for 20 min before treatment with IGFBP-2 for 30 min. ^∗^*P* < 0.05 and ^∗∗∗^*P* < 0.001. Insulin, IGF-1, and IGFBP-2 significantly increase TBC1D1 Ser237 phosphorylation (d) and GLUT-4 translocation (e). ^∗^*P* < 0.05, ^∗∗^*P* < 0.01, and ^∗∗∗^*P* < 0.001 versus control. Each experiment was performed with three technical replicates and total number of experiments was three. The glucose uptake values are percentage of the controls. The results are presented as mean ± SEM.

We further investigated the impact of IGFBP-2 on Akt and AMPK phosphorylation as well as on GLUT-4 translocation. As expected, insulin and IGF-1 significantly (*P* < 0.001) upregulated Akt phosphorylation in treated 3T3-L1 adipocytes. Similarly, IGFBP-2 induced a noticeable increase in Akt phosphorylation in 3T3-L1 adipocytes treated for either 30 min (*P* < 0.05) or 24 h (*P* < 0.01) (Figure 4(a)).

IGF-1 significantly (*P* < 0.001) increased, whereas insulin failed to induce (*P* > 0.05) AMPK phosphorylation in 3T3-L1 adipocytes (Figure 4(b)). Similarly, treatment of adipocytes with IGFBP-2 for either 30 min or 24 h induced a significant (*P* < 0.001) increase in AMPK phosphorylation.

To further confirm the involvement of AMPK phosphorylation in IGFBP-2-stimulated glucose uptake, adipocytes were treated with IGFBP-2 with or without previous incubation with the AMPK inhibitor Compound C. Treatment of the 3T3-L1 adipocytes with IGFBP-2 significantly (*P* < 0.01) increased glucose uptake, an effect that was significantly (*P* < 0.001) abolished by Compound C (Figure 3(c)).

Insulin and IGF-1 stimulation increased TBC1D1 phosphorylation significantly (*P* < 0.05) when compared with the control adipocytes (Figure 4(d)). Similarly, treatment of the 3T3-L1 adipocytes with IGFBP-2 for either 30 min or 24 h induced a significant (*P* < 0.05) increase in TBC1D1 phosphorylation (Figure 4(d)).

GLUT-4 translocation was assessed by subcellular fractionation followed by Western blotting. Treatment of the adipocytes with insulin significantly (*P* < 0.01) stimulated GLUT-4 translocation from the cytoplasm to the plasma membrane. IGF-1 was also able to significantly (*P* < 0.05) stimulate GLUT-4 translocation. Similarly, IGFBP-2 induced a significant (*P* < 0.05) increase in GLUT-4 translocation in treated 3T3-L1 adipocytes (Figure 4(e)).

### 3.5. IGFBP-2 Stimulates PKC*ζ*/*λ* Thr410/403 Phosphorylation in 3T3-L1 Adipocytes

**Figure 5:**
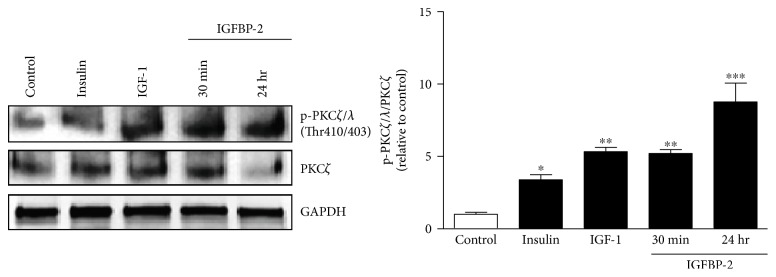
IGFBP-2 stimulates PKC*ζ*/*λ* Thr410/403 phosphorylation in 3T3-L1 adipocytes. Insulin, IGF-1, and IGFBP-2 increase the phosphorylation of PKC*ζ*/*λ*. Each experiment was performed with three technical replicates and total number of experiments was three. The results are presented as mean ± SEM. ^∗^*P* < 0.05, ^∗∗^*P* < 0.01, and ^∗∗∗^*P* < 0.001 versus control.

A significant increase in the phosphorylated levels of the PKC*ζ*/*λ* isoform was seen after stimulation with insulin (*P* < 0.05) or IGF-1 (*P* < 0.01) (Figure 5). Similarly, treatment of the cells with IGFBP-2 induced a significant increase in PKC*ζ*/*λ* phosphorylation after either 30 min (*P* < 0.01) or 24 h (*P* < 0.001) (Figure 5).

## 4. Discussion

Previous studies have indicated the role of IGFBP-2 in adipogenesis and lipogenesis, but its effects on basal glucose uptake and the underlying mechanistic pathways have not yet been addressed. We, herein, provide the first evidence for insulin and IGF-1 independent positive impact of IGFBP-2 on glucose uptake in adipocytes. We further show that the effect of IGFBP-2 on glucose uptake is mediated through the activation of PI3K/Akt and AMPK pathways. Finally, we show that the IGF-1 receptor is neither involved in the IGF-1-induced nor in the IGFBP-2-induced increase in glucose uptake.

Insulin and IGF-1 exerted significant dose-dependent effects on glucose uptake in 3T3-L1 adipocytes. These findings are in agreement with the reports from different previous studies [29–33]. Multiple *in vivo* studies reported the role of IGF-1 in enhancing insulin sensitivity and glucose metabolism. A low-serum level of IGF-1 has been associated with insulin resistance, and treatment with recombinant IGF-1 has been shown to improve insulin sensitivity and glucose metabolism [34, 35]. A study by Arafat et al. [36] revealed that long-term treatment of GH-deficient patients with low-dose GH results in improved insulin sensitivity and enhanced glucose metabolism. This improvement in insulin sensitivity is believed to be mediated by IGF-1, which is secreted as a result of GH stimulation. In another clinical study, IGF-1 combined with IGFBP-3 has been shown to improve insulin sensitivity and to reduce complications associated with insulin resistance in HIV/AIDS patients on antiretroviral therapy [37]. Blocking the insulin receptor with S961 or knocking down the INSR using siRNA significantly reduced basal and insulin-stimulated glucose uptake. The mechanisms behind the effect of S961 on basal glucose uptake in 3T3-L1 are not known so far. However, our results were concordant with previously reported impact of S961 on insulin-stimulated glucose uptake in 3T3-L1 adipocytes [38, 39]. Despite the fact that insulin and IGF-1 have different affinities to INSR and IGF-1R, they are able to stimulate both receptors [40]. However, blocking the IGF-1 receptor using PPP [41] in our present study did not affect the impact of IGF-1 on glucose uptake, whereas blocking or even knocking down the INSR did, pointing to the role of INSR in mediating these IGF-1 effects. In the study of Girnita et al. [41], PPP efficiently blocked IGF-1R activity and reduced phosphorylation of Akt and extracellular signal-regulated kinase 1 and 2 (Erk1/2) in cultured IGF-1R-positive tumor cells. In an *in vitro* kinase assay, PPP did not affect the INSR or compete with ATP [41]. Our findings are also supported by various reports that demonstrated a dramatic increase in INSR and a decrease in IGF-1R during the transition from preadipocytes to adipocytes in the 3T3-L1 cell line [42–45].

IGF-1-dependent and independent effects of IGFBPs on metabolism represent a rapidly growing field of research. IGFBP-1 was reported to inhibit IGF-1-stimulated glucose uptake but not insulin-stimulated glucose uptake in 3T3-L1 adipocytes [31]. IGFBP-3 can lead to insulin resistance in 3T3-L1 adipocytes as reported by Chan et al. [46]. There is increasing evidence for the role of IGFBP-2 in regulating normal metabolism [47]. Low-serum levels of IGFBP-2 are correlated with obesity [22], metabolic syndrome [18], and type 2 diabetes [48], whereas overexpression of IGFBP-2 protects against diabetes and obesity [22, 23]. Roles of IGFBP-2 on metabolism such as inhibition of adipogenesis and lipogenesis [49], enhancing insulin-stimulated glucose uptake in skeletal myotubes [50], and inhibition of preadipocyte differentiation *in vitro* [14] have been reported. However, the effects of IGFBP-2 on basal glucose uptake and the mechanisms underlying its IGF-1-independent role on glucose uptake are not well studied. Here, we reported significantly increased glucose uptake in 3T3-L1 adipocytes treated with 100 nM IGFBP-2. To our knowledge, this is the first report to show the stimulatory effects of IGFBP-2 on basal glucose uptake in adipocytes. In addition, our data showed a nonsignificant effect for short- and long-term treatment with IGFBP-2 on IGF-1 and IGF-1 LR3-stimulated glucose uptake. Adipocytes treated with IGFBP-2 for 24 h followed by 30 min stimulation with IGF-1 showed a trend increase in glucose uptake. Increased basal glucose uptake in control cells incubated with IGFBP-2 for 24 h may explain this increase. However, incubation with IGFBP-2 for 24 h exerted a significant additive effect on the insulin-stimulated glucose uptake which coincides with the study of Yau et al. [50] who reported similar effect for IGFBP-2 in human skeletal muscle cells *in vitro*. It can be postulated that the additive increase in the acute insulin-induced stimulation of glucose uptake after long-term treatment with IGFBP-2 is due to the impact of IGFBP-2 on basal glucose uptake that is likely also mediated through different signaling pathways other than the PI3K/Akt pathway. Moreover, these findings provide a notion that IGFBP-2 binding to IGF-1 does not inhibit IGF-1 from exerting its biological role, at least on glucose uptake *in vitro.*

In addition to its ability to bind and modulate the activity of IGFs, IGFBP-2 can bind to proteoglycans [51] through two heparin-binding domains (HBDs) as well as to integrins through its integrin-binding motif, Gly-Arg-Asp (RGD) [51, 52]. This may explain, at least in part, the IGFR-independent IGFBP-2 activities [49].

Interestingly, neither S961 nor PPP blocked the stimulatory effect of IGFBP-2 on glucose uptake. Moreover, INSR knockdown even increased IGFBP-2-induced increase in glucose uptake. These findings indicate the involvement of other receptors or pathways in IGFBP-2-stimulated glucose uptake in 3T3-L1 adipocytes. This is concordant with the findings of Xi et al. [53], who reported that IGFBP-2 stimulates AMPK via its own receptor.

Signaling via INSR and IGF-1R shares many common signaling pathways at target cells. One of the common pathways in mediating glucose uptake and metabolism is the PI3K pathway [40, 54]. Insulin and IGF-1 are known to stimulate the activity of PI3K by triggering its phosphorylation at specific tyrosine residues by upstream components of the INSR and IGF-1R signaling pathways [55]. In the present study, the PI3K inhibitors, LY294002 and wortmannin, reduced basal and insulin, IGF-1, and even IGFBP-2-stimulated glucose uptake in adipocytes, pointing to the role of PI3K pathway in mediating the IGFBP-2 effect on glucose uptake. We, therefore, investigated the impact of short- and long-term treatment with IGFBP-2 on PI3K phosphorylation in 3T3-L1 adipocytes, using insulin and IGF-1 as controls. As expected, treatment of the adipocytes with either insulin or IGF-1 significantly increased PI3K phosphorylation. Similarly, IGFBP-2 induced marked increase in PI3K phosphorylation after both short- and long-term treatment, confirming the involvement of PI3K activation in mediating IGFBP-2 effects.

Given that IGFBP-2 activates PI3K, we tested its effect on the downstream signaling molecules Akt and AMPK and GLUT-4 translocation. As a result of PI3K activation, insulin and IGF-1 stimulated the phosphorylation of Akt. These findings are in agreement with the studies of Karlsson et al. [56] and Zhang et al. [57]. Moreover, IGF-1 significantly increased AMPK phosphorylation. IGF-1 has been previously shown to stimulate the phosphorylation of AMPK at its alpha subunit [58]. On the other hand, insulin did not affect the level of p-AMPK indicating that insulin mainly uses the PI3K pathway to exert its effects on glucose metabolism. Our findings are in agreement with Shen et al. [59] who clearly showed that insulin does not stimulate AMPK. In the same context, pharmacological activation of AMPK increases glucose uptake in skeletal muscles of subjects with type 2 diabetes [60] by an insulin-independent mechanism [61].

Similarly, IGFBP-2 produced a significant increase in Akt phosphorylation which is attributed to its stimulatory effect on PI3K. Concordant data were reported by Yau et al. [50] in human skeletal muscle cells. The surface proteoglycan receptor-type protein tyrosine phosphatase *β* (RPTP*β*) has been identified as a functionally active cell surface receptor that links IGFBP-2 and the activation of Akt [62]. IGFBP-2 binds RPTP*β* through its HBD, resulting in inhibition of RPTP*β* phosphatase activity and subsequently phosphatase and tensin homolog (PTEN) suppression [62]. PTEN is known to prevent Akt activation by dephosphorylating phosphatidylinositol-3,4,5-triphosphate (PIP3). The study of Shen et al. [62] showed that IGFBP-2^−/−^ mice had increased RPTP*β* activity and impaired Akt activation, changes that were reversed by administration of IGFBP-2.

In addition, both short- and long-term treatment of the adipocytes with IGFBP-2 induced a significant increase in AMPK phosphorylation. IGFBP-2 and IGF-1 have been recently reported by Xi et al. [53] to induce stimulatory effects on AMPK in osteoblasts. Our results were further confirmed through testing the effect of AMPK inhibitor, Compound C, on IGFBP-2-stimulated glucose uptake. Incubation of the adipocytes with Compound C significantly abolished IGFBP-2-induced glucose uptake. Taken together, AMPK activation plays a potential role in mediating IGFBP-2-stimulated glucose uptake in 3T3-L1 adipocytes.

One of the major metabolic changes elicited by AMPK activation is the promotion of glucose uptake [63]. AMPK induces glucose uptake either acutely through GLUT-4 translocation or in the longer term via upregulation of GLUT-4 expression [63]. Here, we show that treatment of the 3T3-L1 adipocytes with IGFBP-2 for 30 min stimulates GLUT-4 translocation to the plasma membrane. This effect is attributed to the ability of IGFBP-2 to activate AMPK. In addition, we show a significant increase in the phosphorylation of the Rab-GAP protein TBC1D1 by IGFBP-2. Therefore, the mechanism underlying the IGFBP-2 impact on GLUT-4 translocation and the subsequent promotion of glucose uptake involves the phosphorylation of TBC1D1 at least partly through AMPK-pathway activation. This effect is similar to the complementary regulation of TBC1D1 by insulin and AMPK activators [64, 65]. Increased TBC1D1 phosphorylation and GLUT-4 translocation by IGFBP-2 could also be directly mediated by Akt activation. In the skeletal muscle of rodents, Akt phosphorylates TBC1D1 [66] which promotes the hydrolysis of guanosine-5′-triphosphate on GLUT-4-containing vesicles [67].

The atypical protein kinase PKC*ζ*/*λ*/GLUT-4 is another signaling pathway we thought to have a role in mediating the positive effect of IGFBP-2 on glucose uptake in adipocytes. In our study, insulin, IGF-1, and IGFBP-2 induced a significant increase in PKC*ζ*/*λ* phosphorylation. Since, PKC*ζ*/*λ* is dependent on PI3K activation [29, 68], it was expected to be activated in adipocytes treated with insulin, IGF-1, and IGFBP-2 because of their ability to activate PI3K. Following activation, PI3K signaling diverges into Akt-dependent and PKC*ζ*/*λ*-mediated pathways [69]. PKC*ζ*/*λ* is known to play little or no role in mediating insulin effects on glucose uptake in 3T3-L1 adipocytes [70], which may explain the IGFBP-2-induced additive increase in the insulin-induced glucose uptake after long-term treatment in our present study. However, further studies using inhibitors or gene silencing are needed to explore the precise involvement of PKC*ζ*/*λ* in mediating the IGFBP-2-induced increase in glucose uptake and GLUT-4 translocation. One of the limitations of our study was the IGFBP-2 concentrations used to elicit a significant impact on glucose uptake. IGFBP-2 increased glucose uptake at concentrations 7–10-fold higher than those described in humans. Therefore, further *in vivo* studies are needed to explore the precise impact of physiological concentrations of IGFBP-2 on glucose utilization in humans.

In summary, this study shows that IGFBP-2 stimulates glucose uptake in 3T3-L1 adipocytes and that synergistic activation of Akt and AMPK mediates the modulatory effect of IGFBP-2. The PI3K/PKC*ζ*/*λ*/GLUT-4 signaling is here shown to mediate the IGFBP-2-induced increase in glucose uptake. Furthermore, we showed that IGFBP-2-induced glucose uptake is independent of its binding to IGF-1, INSR, and IGF-1R. Our findings highly strengthen the potential and novel role for IGFBP-2 in glucose metabolism.

## Figures and Tables

**Table 1:** Primers used for qRT-PCR.

Gene	Sequence (5′-3′)
*INSR*	F: GTACTGGGAGAGGCAAGCAG
	R: ACTGGCCGAGTCGTCATACT

*36B4*	F: TCATCCAGCAGGTGTTTGACA
	R: GGCACCGAGGCAACAGTT

**Table 2:** List of antibodies used.

Antibody	Species	Supplier	Catalog number
Anti-Phospho-AMPK*α* (Thr172)	Rabbit	Cell Signaling Technology	2535
Anti-AMPK*α*	Rabbit	Cell Signaling Technology	2532
Anti-Phospho-Akt (Ser473)	Rabbit	Cell Signaling Technology	9271
Anti-Akt	Rabbit	Cell Signaling Technology	9272
Anti-Phospho-(Tyr) p85 PI3K	Rabbit	Cell Signaling Technology	3821
Anti-PI3K p85	Rabbit	Cell Signaling Technology	4292
Anti-Phospho-TBC1D1 (Ser237)	Rabbit	Millipore	07–2268
Anti-TBC1D1	Rabbit	Cell Signaling Technology	5929
Anti-Phospho-PKC*ζ*/*λ* (Thr410/403)	Rabbit	Cell Signaling Technology	9378
Anti-PKC*ζ*	Mouse	Santa Cruz Biotechnology	SC-17781
Anti-GLUT-4	Rabbit	Sigma	G4173
Anti-Na^+^, K^+^-ATPase	Rabbit	Cell Signaling Technology	3010
Anti-GAPDH	Rabbit	Cell Signaling Technology	2118
Goat anti-rabbit IgG HRP-linked	Goat	Cell Signaling Technology	4074
Horse anti-mouse IgG HRP-linked	Horse	Cell Signaling Technology	4076

## References

[1] Rinderknecht E., Humbel R. E. (1978). The amino acid sequence of human insulin-like growth factor I and its structural homology with proinsulin. *Journal of Biological Chemistry*.

[2] Smith P. J., Wise L. S., Berkowitz R., Wan C., Rubin C. S. (1988). Insulin-like growth factor-I is an essential regulator of the differentiation of 3T3-L1 adipocytes. *Journal of Biological Chemistry*.

[3] O’Dell S. D., Day I. N. M. (1998). Insulin-like growth factor II (IGF-II). *International Journal of Biochemistry & Cell Biology*.

[4] Monzavi R., Cohen P. (2002). IGFs and IGFBPs: role in health and disease. *Best Practice & Research Clinical Endocrinology & Metabolism*.

[5] Skottner A. (2012). Biosynthesis of growth hormone and insulin-like growth factor-I and the regulation of their secretion. *The Open Endocrinology Journal*.

[6] Butler S. T., Marr A. L., Pelton S. H., Radcliff R. P., Lucy M. C., Butler W. R. (2003). Insulin restores GH responsiveness during lactation-induced negative energy balance in dairy cattle: effects on expression of IGF-I and GH receptor 1A. *Journal of Endocrinology*.

[7] Rhoads R. P., Kim J. W., Leury B. J. (2004). Insulin increases the abundance of the growth hormone receptor in liver and adipose tissue of periparturient dairy cows. *The Journal of Nutrition*.

[8] Clemmons D. R. (2004). The relative roles of growth hormone and IGF-1 in controlling insulin sensitivity. *The Journal of Clinical Investigation*.

[9] Bach L. A., Headey S. J., Norton R. S. (2005). IGF-binding proteins – the pieces are falling into place. *Trends in Endocrinology & Metabolism*.

[10] Kutsukake M., Ishihara R., Momose K. (2008). Circulating IGF-binding protein 7 (IGFBP7) levels are elevated in patients with endometriosis or undergoing diabetic hemodialysis. *Reproductive Biology and Endocrinology*.

[11] Clemmons D. R. (2016). Role of IGF binding proteins in regulating metabolism. *Trends in Endocrinology & Metabolism*.

[12] Russo V. C., Gluckman P. D., Feldman E. L., Werther G. A. (2005). The insulin-like growth factor system and its pleiotropic functions in brain. *Endocrine Reviews*.

[13] Mohan S., Baylink D. J. (2002). IGF-binding proteins are multifunctional and act via IGF-dependent and -independent mechanisms. *Journal of Endocrinology*.

[14] Wheatcroft S. B., Kearney M. T. (2009). IGF-dependent and IGF-independent actions of IGF-binding protein-1 and -2: implications for metabolic homeostasis. *Trends in Endocrinology & Metabolism*.

[15] Boney C. M., Moats-Staats B. M., Stiles A. D., D’Ercole A. J. (1994). Expression of insulin-like growth factor-I (IGF-I) and IGF-binding proteins during adipogenesis. *Endocrinology*.

[16] Hoeflich A., Reisinger R., Lahm H. (2001). Insulin-like growth factor-binding protein 2 in tumorigenesis: protector or promoter?. *Cancer Research*.

[17] Fukushima T., Kataoka H. (2007). Roles of insulin-like growth factor binding protein-2 (IGFBP-2) in glioblastoma. *Anticancer Research*.

[18] Heald A. H., Kaushal K., Siddals K. W., Rudenski A. S., Anderson S. G., Gibson J. M. (2006). Insulin-like growth factor binding protein-2 (IGFBP-2) is a marker for the metabolic syndrome. *Experimental and Clinical Endocrinology & Diabetes*.

[19] Li Z., Picard F. (2010). Modulation of IGFBP2 mRNA expression in white adipose tissue upon aging and obesity. *Hormone and Metabolic Research*.

[20] Nam S. Y., Lee E. J., Kim K. R. (1997). Effect of obesity on total and free insulin-like growth factor (IGF)-1, and their relationship to IGF-binding protein (BP)-1, IGFBP-2, IGFBP-3, insulin, and growth hormone. *International Journal of Obesity*.

[21] Claudio M., Benjamim F., Riccardo B., Massimiliano C., Francesco B., Luciano C. (2010). Adipocytes IGFBP-2 expression in prepubertal obese children. *Obesity*.

[22] Wheatcroft S. B., Kearney M. T., Shah A. M. (2007). IGF-binding protein-2 protects against the development of obesity and insulin resistance. *Diabetes*.

[23] Hedbacker K., Birsoy K., Wysocki R. W. (2010). Antidiabetic effects of IGFBP2, a leptin-regulated gene. *Cell Metabolism*.

[24] Yau S. W., Russo V. C., Clarke I. J., Werther G. A., Sabin M. A. (2013). IGFBP-2 enhances insulin signalling and glucose uptake in human skeletal myotubes. *Obesity Research & Clinical Practice*.

[25] Woody S., Stall R., Ramos J., Patel Y. M. (2013). Regulation of myosin light chain kinase during insulin-stimulated glucose uptake in 3T3-L1 adipocytes. *PLoS One*.

[26] Yamamoto N., Ueda M., Sato T. (2011). Measurement of glucose uptake in cultured cells. *Current Protocols in Pharmacology*.

[27] Laajoki L. G., Francis G. L., Wallace J. C., Carver J. A., Keniry M. A. (2000). Solution structure and backbone dynamics of long-[Arg^3^]insulin-like growth factor-I. *Journal of Biological Chemistry*.

[28] Ramlal T., Sarabia V., Bilan P. J., Klip A. (1988). Insulin-mediated translocation of glucose transporters from intracellular membranes to plasma membranes: sole mechanism of stimulation of glucose transport in L6 muscle cells. *Biochemical and Biophysical Research Communications*.

[29] Kotani K., Ogawa W., Matsumoto M. (1998). Requirement of atypical protein kinase Cλ for insulin stimulation of glucose uptake but not for Akt activation in 3T3-L1 adipocytes. *Molecular and Cellular Biology*.

[30] Rice K. M., Garner C. W. (1999). IGF-I regulates IRS-1 expression in 3T3-L1 adipocytes. *Biochemical and Biophysical Research Communications*.

[31] Siddals K. W., Westwood M., Gibson J. M., White A. (2002). IGF-binding protein-1 inhibits IGF effects on adipocyte function: implications for insulin-like actions at the adipocyte. *Journal of Endocrinology*.

[32] Takahashi M., Takahashi Y., Takahashi K. (2008). Chemerin enhances insulin signaling and potentiates insulin-stimulated glucose uptake in 3T3-L1 adipocytes. *FEBS Letters*.

[33] Berenguer M., Zhang J., Bruce M. C. (2011). Dimethyl sulfoxide enhances GLUT4 translocation through a reduction in GLUT4 endocytosis in insulin-stimulated 3T3-L1 adipocytes. *Biochimie*.

[34] Yuen K. C., Dunger D. B. (2006). Impact of treatment with recombinant human GH and IGF-I on visceral adipose tissue and glucose homeostasis in adults. *Growth Hormone & IGF Research*.

[35] Yuen K. C., Dunger D. B. (2007). Therapeutic aspects of growth hormone and insulin-like growth factor-I treatment on visceral fat and insulin sensitivity in adults. *Diabetes, Obesity and Metabolism*.

[36] Arafat A. M., Möhlig M., Weickert M. O., Schöfl C., Spranger J., Pfeiffer A. F. (2010). Improved insulin sensitivity, preserved beta cell function and improved whole-body glucose metabolism after low-dose growth hormone replacement therapy in adults with severe growth hormone deficiency: a pilot study. *Diabetologia*.

[37] Rao M. N., Mulligan K., Tai V. (2010). Effects of insulin-like growth factor (IGF)-I/IGF-binding protein-3 treatment on glucose metabolism and fat distribution in human immunodeficiency virus-infected patients with abdominal obesity and insulin resistance. *The Journal of Clinical Endocrinology & Metabolism*.

[38] Schäffer L., Brand C. L., Hansen B. F. (2008). A novel high-affinity peptide antagonist to the insulin receptor. *Biochemical and Biophysical Research Communications*.

[39] Knudsen L., Hansen B. F., Jensen P. (2012). Agonism and antagonism at the insulin receptor. *PLoS One*.

[40] Siddle K. (2012). Molecular basis of signaling specificity of insulin and IGF receptors: neglected corners and recent advances. *Frontiers in Endocrinology*.

[41] Girnita A., Girnita L., del Prete F., Bartolazzi A., Larsson O., Axelson M. (2004). Cyclolignans as inhibitors of the insulin-like growth factor-1 receptor and malignant cell growth. *Cancer Research*.

[42] Reed B. C., Kaufmann S. H., Mackall J. C., Student A. K., Lane M. D. (1977). Alterations in insulin binding accompanying differentiation of 3T3-L1 preadipocytes. *Proceedings of the National Academy of Sciences of the United States of America*.

[43] Boney C. M., Smith R. M., Gruppuso P. A. (1998). Modulation of insulin-like growth factor I mitogenic signaling in 3T3-L1 preadipocyte differentiation. *Endocrinology*.

[44] Hong S., Huo H., Xu J., Liao K. (2004). Insulin-like growth factor-1 receptor signaling in 3T3-L1 adipocyte differentiation requires lipid rafts but not caveolae. *Cell Death and Differentiation*.

[45] Boucher J., Tseng Y. H., Kahn C. R. (2010). Insulin and insulin-like growth factor-1 receptors act as ligand-specific amplitude modulators of a common pathway regulating gene transcription. *Journal of Biological Chemistry*.

[46] Chan S. S., Twigg S. M., Firth S. M., Baxter R. C. (2005). Insulin-like growth factor binding protein-3 leads to insulin resistance in adipocytes. *The Journal of Clinical Endocrinology & Metabolism*.

[47] Sabin M. A., Russo V. C., Azar W. J., Yau S. W., Kiess W., Werther G. A. (2011). IGFBP-2 at the interface of growth and metabolism--implications for childhood obesity. *Pediatric Endocrinology Reviews*.

[48] Frystyk J., Skjaerbaek C., Vestbo E., Fisker S., Orskov H. (1999). Circulating levels of free insulin-like growth factors in obese subjects: the impact of type 2 diabetes. *Diabetes/Metabolism Research and Reviews*.

[49] Yau S. W., Russo V. C., Clarke I. J., Dunshea F. R., Werther G. A., Sabin M. A. (2015). IGFBP-2 inhibits adipogenesis and lipogenesis in human visceral, but not subcutaneous, adipocytes. *International Journal of Obesity*.

[50] Yau S. W., Russo V. C., Werther G. A., Sabin M. A. (2014). IGFBP-2 enhances insulin signalling pathways in human skeletal muscle by cell surface binding – an IGF-independent process?. *Endocrine Society’s 96th Annual Meeting and Expo*.

[51] Russo V. C., Schütt B. S., Andaloro E. (2005). Insulin-like growth factor binding protein-2 binding to extracellular matrix plays a critical role in neuroblastoma cell proliferation, migration, and invasion. *Endocrinology*.

[52] Schutt B. S., Langkamp M., Rauschnabel U., Ranke M. B., Elmlinger M. W. (2004). Integrin-mediated action of insulin-like growth factor binding protein-2 in tumor cells. *Journal of Molecular Endocrinology*.

[53] Xi G., Rosen C. J., Clemmons D. R. (2016). IGF-I and IGFBP-2 stimulate AMPK activation and autophagy, which are required for osteoblast differentiation. *Endocrinology*.

[54] Siddle K. (2011). Signalling by insulin and IGF receptors: supporting acts and new players. *Journal of Molecular Endocrinology*.

[55] Dupont J., LeRoith D. (2001). Insulin and insulin-like growth factor I receptors: similarities and differences in signal transduction. *Hormone Research in Pædiatrics*.

[56] Karlsson H. K., Zierath J. R., Kane S., Krook A., Lienhard G. E., Wallberg-Henriksson H. (2005). Insulin-stimulated phosphorylation of the Akt substrate AS160 is impaired in skeletal muscle of type 2 diabetic subjects. *Diabetes*.

[57] Zhang Y., Liu Y., Li X. (2013). Effects of insulin and IGF-I on growth hormone- induced STAT5 activation in 3T3-F442A adipocytes. *Lipids in Health and Disease*.

[58] Suzuki A., Kusakai G., Kishimoto A. (2004). IGF-1 phosphorylates AMPK-*α* subunit in ATM-dependent and LKB1-independent manner. *Biochemical and Biophysical Research Communications*.

[59] Shen Y., Honma N., Kobayashi K. (2014). Cinnamon extract enhances glucose uptake in 3T3-L1 adipocytes and C2C12 myocytes by inducing LKB1-AMP-activated protein kinase signaling. *PLoS One*.

[60] Koistinen H. A., Galuska D., Chibalin A. V. (2003). 5-amino-imidazole carboxamide riboside increases glucose transport and cell-surface GLUT4 content in skeletal muscle from subjects with type 2 diabetes. *Diabetes*.

[61] Hayashi T., Hirshman M. F., Kurth E. J., Winder W. W., Goodyear L. J. (1998). Evidence for 5′ AMP-activated protein kinase mediation of the effect of muscle contraction on glucose transport. *Diabetes*.

[62] Shen X., Xi G., Maile L. A., Wai C., Rosen C. J., Clemmons D. R. (2012). Insulin-like growth factor (IGF) binding protein 2 functions coordinately with receptor protein tyrosine phosphatase *β* and the IGF-I receptor to regulate IGF-I-stimulated signaling. *Molecular and Cellular Biology*.

[63] McGee S. L., van Denderen B. J., Howlett K. F. (2008). AMP-activated protein kinase regulates GLUT4 transcription by phosphorylating histone deacetylase 5. *Diabetes*.

[64] Chen S., Murphy J., Toth R., Campbell D. G., Morrice N. A., Mackintosh C. (2008). Complementary regulation of TBC1D1 and AS160 by growth factors, insulin and AMPK activators. *Biochemical Journal*.

[65] Pehmoller C., Treebak J. T., Birk J. B. (2009). Genetic disruption of AMPK signaling abolishes both contraction- and insulin-stimulated TBC1D1 phosphorylation and 14-3-3 binding in mouse skeletal muscle. *American Journal of Physiology - Endocrinology and Metabolism*.

[66] Taylor E. B., An D., Kramer H. F. (2008). Discovery of TBC1D1 as an insulin-, AICAR-, and contraction-stimulated signaling nexus in mouse skeletal muscle. *Journal of Biological Chemistry*.

[67] Sano H., Kane S., Sano E. (2003). Insulin-stimulated phosphorylation of a Rab GTPase-activating protein regulates GLUT4 translocation. *Journal of Biological Chemistry*.

[68] Ishiki M., Klip A. (2005). Minireview: recent developments in the regulation of glucose transporter-4 traffic: new signals, locations, and partners. *Endocrinology*.

[69] Bandyopadhyay G., Standaert M. L., Sajan M. P. (1999). Dependence of insulin-stimulated glucose transporter 4 translocation on 3-phosphoinositide-dependent protein kinase-1 and its target threonine-410 in the activation loop of protein kinase C-*ζ*. *Molecular Endocrinology*.

[70] Tsuru M., Katagiri H., Asano T. (2002). Role of PKC isoforms in glucose transport in 3T3-L1 adipocytes: insignificance of atypical PKC. *American Journal of Physiology - Endocrinology and Metabolism*.

